# Feedback Regimes of LFI Sensors: Experimental Investigations

**DOI:** 10.3390/s22229001

**Published:** 2022-11-21

**Authors:** Karl Bertling, Xiaoqiong Qi, Thomas Taimre, Yah Leng Lim, Aleksandar D. Rakić

**Affiliations:** 1 School of Information Technology and Electrical Engineering, The University of Queensland, Brisbane, QLD 4072, Australia; 2 School of Mathematics and Physics, The University of Queensland, Brisbane, QLD 4072, Australia

**Keywords:** laser feedback interferometry, self-mixing, laser diode

## Abstract

In this article, we revisit the concept of optical feedback regimes in diode lasers and explore each regime experimentally from a somewhat unconventional point of view by relating the feedback regimes to the laser bias current and its optical feedback level. The results enable setting the operating conditions of the diode laser in different applications requiring operation in different feedback regimes. We experimentally explored and theoretically supported this relationship from the standard Lang and Kobayashi rate equation model for a laser diode under optical feedback. All five regimes were explored for two major types of laser diodes: inplane lasers and vertical-cavity surface emitting lasers. For both lasers, we mapped the self-mixing strength vs. drive current and feedback level, observed the differences in the shape of the self-mixing fringes between the two laser architectures and a general simulation, and monitored other parameters of the lasers with changing optical feedback.

## 1. Introduction

Laser feedback interferometry (LFI) is a technique that exploits the remarkably universal self-mixing (SM) phenomenon, and it was demonstrated with a wide variety of lasers for sensing and imaging applications [1,2,3], including the measurement of vibration, displacement, and distance [4,5,6], velocity and flow [7,8,9], the propagation of elastic waves [10], the complex material properties of external targets in both the far field [11,12,13] and near field [14,15], and for biomedical imaging [16,17]. In a typical LFI architecture, light emitted from a laser cavity is transmitted through an external cavity and reflected by an external target. The reflected light imprinted with target information is reinjected into the laser cavity where it mixes with the intracavity electric field. This nonlinear mixing process results in measurable perturbations of laser parameters that contain information about the external target. The affected laser parameters include threshold gain, lasing frequencies, optical output power, and laser terminal voltage. Compared with monitoring an SM signal through the optical power through the use of a photodiode (PD), monitoring the voltage across laser terminals removes the need for an additional detector [18,19,20], which is very attractive for lasers operating at wavelengths that lack effective detectors, such as for terahertz (THz) quantum cascade lasers (QCLs) [21]. LFI sensing is a coherent sensing technique that can capture the amplitude and phase information of the target. The characteristics of LFI systems have been comprehensively investigated in past years, including the effects of the injection current and temperature on the signal strength of SM signals in single-mode laser diodes (LDs) [22], the pulsed-mode operation of LFI sensors by using THz QCLs [23], and the detection sensitivity of SM signals in a single-mode THz QCL [24]. In addition, SM signals in multiple longitudinal mode Fabry–Perot LDs and multiple transverse modes in vertical-cavity surface-emitting lasers (VCSELs) were investigated [25,26]. Recently, LFI sensors by using multimodal THz QCLs were demonstrated for measuring emission spectrum and gas concentrations at multiple fingerprint frequencies [27,28].

**Figure 1:**
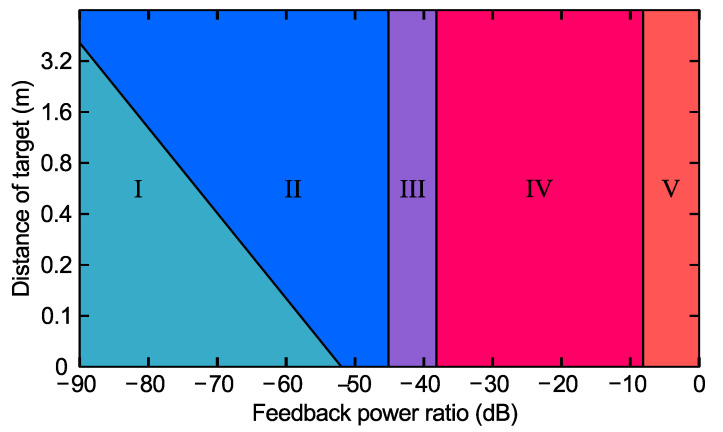
Five classical regimes of optical feedback in a typical laser diode.

Semiconductor lasers are sensitive to external optical feedback, under which they exhibit rich nonlinear dynamics [29,30,31]. Depending on the magnitude of optical feedback and the external cavity length, five regimes of operation were well-defined, and the transitions between them are easily identified in LDs [32,33,34,35,36,37,38,39,40] (see Figure 1). Narrowing or broadening the emission spectrum depending on the phase of the feedback was observed in Regime I with the lowest levels of feedback. An equivalent stimulus to the displacement of the external cavity creates interferograms that have a sinusoidal shape and a period of λ/2 of equivalent displacement [3,41]. The apparent frequency splitting of the emission line arising from rapid external cavity mode hopping was observed in Regime II, with a moderate level of feedback and stimulus, creating an interferogram that became nonlinear, and a transition from sinusoidal to sawtooth-like [42,43,44]. As the feedback was increased further, mode hopping was suppressed, and the laser operated on a single narrow line; this refers to Regime III. Interferograms in this regime transition from sawtooth-like, slowly losing fringes until the result directly mimics the behaviour of the stimulus [5,45]. Frequency spitting at the relaxation oscillation frequency of the laser appears and grows as the feedback increases, and the laser emission line eventually broadens to as much as 50 GHz for a 1.5 µm DFB laser with all feedback phases in Regime IV, which is called coherence collapse (and any interferometric information is lost). The laser operates as a long external cavity laser with single longitudinal mode and narrow line width for all phases of the feedback in Regime V, with the highest levels of feedback usually being greater than −10 dB [32], with stimulus again being mimicked by the laser response. The diagram of feedback regimes in semiconductor lasers was revisited by introducing short and long external cavity lengths, and coherence length in [33], where noteworthy applications of each regime were also discussed, particularly the SM regime (Regimes I and II). The intrinsic stability of midinfrared and THz QCLs against optical feedback was studied in [46,47]. The absence of a reported coherence collapse or other continuous-wave instabilities typical of LDs was observed, possibly due to the high value of the photon-to-carrier-lifetime ratio and the negligible line-width enhancement factor of QCLs. However, all five regimes were identified experimentally in midinfrared QCLs with optical spectrum analysis [48]. The chaotic operation of midinfrared QCLs was also observed in [49,50]. Recent observations in THz QCLs also showed fringe loss associated with Regime III [51,52] and the support of external cavity oscillations resulting from self-pulsations due to higher feedback levels [53,54].

In this work, classical optical feedback regimes are explored from a different point of view by mapping the space of the bias current and optical feedback level. Such a map allows for determining the practical operation conditions of the laser for different applications. The diagram of feedback regimes in the bias current and optical feedback domain are experimentally explored and theoretically supported from the standard Lang and Kobayashi rate equation model for an LD under optical feedback. All five regimes are captured in two different types of LDs: a VCSEL and a DFB laser. The LFI waveforms at different regimes and the sensitivity at SM Regimes (I and II) are also compared with simulation results obtained from the rate equation model, comparing favorably.

## 2. Theoretical Model of Laser Feedback Interferometry Signals

The theoretical model that used to simulate LFI waveforms and predict the sensitivity of the LFI sensors is described in this section. The fundamental model for a semiconductor laser with a single longitudinal mode experiencing optical feedback is that of Lang and Kobayashi [55]. In an LFI sensor system, light reflected from the target is reinjected into the laser cavity, where it mixes with an intracavity electric field, and results in measurable perturbations of optical output power and laser terminal voltage. We simulated the SM signal through terminal voltage instead of optical output power to align with the experimental observations appearing in subsequent sections. The laser terminal voltage $V_{\mathrm{Terminal}}\left(t\right)$ for a laser diode can be modelled as follows [56,57]:

(1) $$V_{\mathrm{Terminal}}\left(t\right)=\frac{2k_{B}T}{q}\ln\left(\frac{N(t)}{N_{i}}\right)\;,$$

where N(t) is the carrier density of the active region, and $N_{i}$ is the intrinsic carrier density of the active region. The meaning of other symbols and typical values used in the simulation are summarized in the Appendix Table A1. In an LFI system, when the frequency of system stimuli such as perturbations from the target movement are slow relative to the natural laser relaxation frequency and the natural resonant frequency of the external cavity, the dynamic rate equation model can be reduced to a temporal steady state. Steady-state carrier density $N_{s}$ can be described with the the solution of the Lang and Kobayashi rate equations in steady state, as shown below:

(2) $$\begin{array}{c} N_{s}=N_{\mathrm{tr}}+\frac{1-2\tilde{\kappa }\tau_{p}\cos\phi_{\mathrm{FB}}}{\Gamma v_{g}a\tau_{p}}=N_{0}\left(1-\frac{2\tilde{\kappa }}{N_{0}\Gamma v_{g}a}\cos\phi_{\mathrm{FB}}\right)\;, \end{array}$$

where $N_{0}=N_{\mathrm{tr}}+\frac{1}{\Gamma v_{g}a\tau_{p}}$ is the steady-state solution of the carrier density in the absence of optical feedback, $\phi_{\mathrm{FB}}$ is the steady-state phase under optical feedback, $N_{\mathrm{tr}}$ is the transparency carrier density, Γ is the confinement factor, $v_{g}$ is the group velocity, *a* is the differential gain, and $\tau_{p}$ is the photon lifetime. Term $\tilde{\kappa }=\epsilon \left(1-R_{2}\right)\sqrt{R/R_{2}}/\tau_{\mathrm{in}}$ is the feedback coupling rate, where ϵ is the coupling ratio of the reinjected light into the laser cavity, $R_{2}$ and *R* are the reflection coefficients of the laser facet from which the photons leave the laser cavity and the external target, respectively, and $\tau_{\mathrm{in}}$ is the intracavity round-trip time. Term $\phi_{\mathrm{FB}}$, in turn, satisfies the excess phase equation:

(3) $$\begin{array}{c} \phi_{\mathrm{FB}}-\phi_{s}+C\sin\left(\phi_{\mathrm{FB}}+\arctan\alpha \right)=0\;, \end{array}$$

where $\phi_{s}$ is defined as $\omega_{s}\tau_{\mathrm{ext}}$, which describes the phase accumulated on transmission through the external cavity if the laser were not experiencing optical feedback, *C* is the feedback-level parameter defined as $C=\tilde{\kappa }\tau_{\mathrm{ext}}\sqrt{1+\alpha^{2}}$ with $\tau_{\mathrm{ext}}$ being the external cavity round-trip time, and α the line-width enhancement factor. Steady-state terminal voltage is used to model the SM waveforms and their dynamic range at various attenuation values in this paper from regimes I–III in order to carry out a direct comparison with the experimental results.

If we rewrote the steady-state solution of the carrier density as $N_{s}=N_{0}\left(1+\beta \cos\phi_{\mathrm{FB}}\right)$ and compared it with Equation (2), we would have $\beta =-\frac{2\tilde{\kappa }}{N_{0}\Gamma v_{g}a}$, which is different from β for the optical power [22].

## 3. Experimental Setup

**Figure 2:**
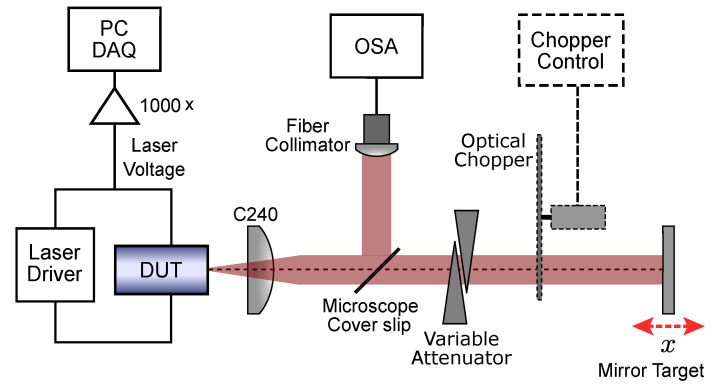
Experimental setup used to evaluate SM in the tested lasers.

The experimental setup is shown in Figure 2, with the device under test (DUT) representing the position of the two tested lasers. The 850 nm VCSEL (Litrax LX-VCS-850-T101) was operated at 35 ${}^{\circ }$C ($I_{\mathrm{th}}=3.8$ mA), and the 852 nm DFB (Eagleyard Photonics EYP-DFB-0852-00150-1500-SOT02-0000) was operated at 25 ${}^{\circ }$C ($I_{\mathrm{th}}=30$ mA). In both cases, the laser beam was collimated using a lens (C240 Thorlabs) onto a mirror mounted onto a precision piezoelectric actuator stage (PI P-752 11C) driven by a piezo controller (PI E-665 CR). The efficacy of the collimation was tested using a shearing plate that allowed for precise collimation (deviation from true collimation produced markedly different results). A variable dual-wedge attenuator was used to control the feedback levels (Newport 925B), and a microscope cover slip was use to sample the beam (6% reflection at 850 nm) and fed into a optical-spectrum analyser (OSA) (Agilent 86140B). Laser drive current and temperature were controlled with a combined laser driver temperature controller (Arroyo Instruments 6305 ComboSource). The signal was obtained from laser terminal voltage passed through a custom built differential input variable gain amplifier, and was averaged 16 times for all measurements.

Three experiments were performed. (1) The mirror target was displaced and current swept, (2) static mirror with optical chopper in place, and the (3) static mirror and optical spectrum were observed. These were then repeated for a wide range of feedback conditions that were adjusted via the variable attenuator. The variable attenuator allowed for a range of round-trip attenuation from −1 to −60 dB, allowing for showcasing all feedback regimes (Experiments (2) and (3) were performed a fixed current that would allow for the greatest visualisation of feedback effects). Both lasers were nominally single-mode in the entire sweep range and had constant linear optical power vs. current characteristics (VCSEL sweep 0 to 12 mA, DFB 0 to 100 mA). These three experiments allowed for us to observe several phenomena with respect to changes in the optical feedback: (1) changes in fringe strength (in our case, the peak-to-peak of the SM waveform) and in the morphology of the SM signal with periodic motion (as in [1,58,59,60,61,62]), (2) difference between reflection-based feedback (with the optical chopper changing the optical path between full and no feedback), which informs how β changes (as exploited in [4,56,63,64,65,66]), and lastly (3) any major changes with the laser spectrum (behaviour observed in [25,67]).

## 4. Results and Discussion

**Figure 3:**
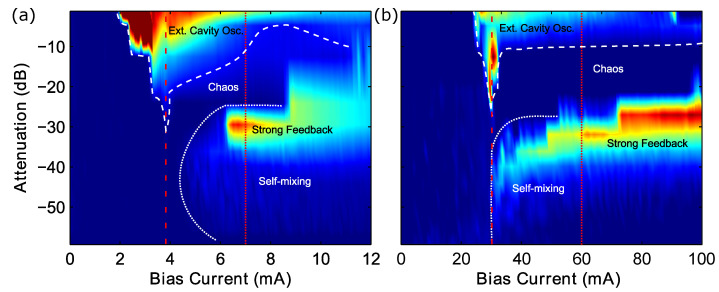
Bias current vs. attenuation vs. amplitude (peak-to-peak of SM displacement signal). (dashed line = Ith, dotted line = bias current, where representative waveforms and spectra were measured). (**a**) 850 nm VCSEL; (**b**) 852 nm DFB.

From the first experiment, we obtained a mapping of the SM signal strength in terms of the peak-to-peak of the fringes/waveform vs. the drive current of the laser vs. the feedback level (controlled by the attenuation). These mappings show a wide range of phenomena for both the VCSEL (Figure 3a) and the DFB laser (Figure 3b). Both show the effects of the SM phenomenon from low to strong feedback, the descent into chaos, and the establishment of external cavity modes (and external cavity operation). The precise alignment of the external cavity allowed for these observations to be observed at nearly the same coupling conditions (dBs) reported for the theoretical evaluations of the feedback regimes. Notable differences were in the onset of the external cavity modes/operation, and when the SM signal started at around the threshold. The VCSEL showed a pronounced delay after the threshold in the onset of SM (also observed in [68]), while the DFB seemed to start immediately at the threshold [22]. Both lasers showed a decrease in the threshold when establishing external cavity modes/operation with the extra light reinjected into the laser cavity from the external mirror, essentially reducing the required current for lasing to occur.

**Figure 4:**
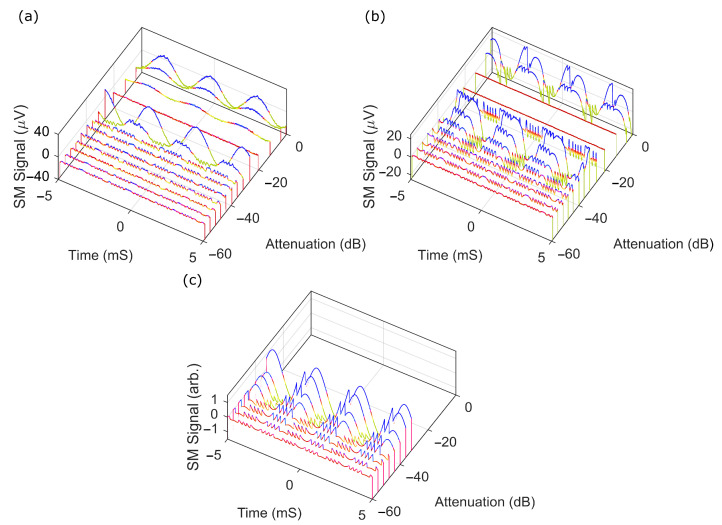
Self-mixing signal for different feedback levels (attenuation in dB) for harmonic displacement target. (**a**) VCSEL at a bias current of 7 mA and laser temperature of 35 ${}^{\circ }$C; (**b**) DFB at a bias current of 60 mA and laser temperature of 25 ${}^{\circ }$C; (**c**) simulated DFB.

Some of these observed differences could be seen in the waveforms obtained at these different conditions. Figure 4 shows an exemplary set of SM waveforms as the middle operating point for each laser vs. changes in the feedback level. For the VCSEL, the waveforms went through all the classical regimes (I–V) with almost textbook clarity. It was easy to observe (I) weak, (II) moderate, and (III) strong feedback (including complete fringe loss) giving way to (IV) chaos and then (V) external cavity oscillations. The DFB, however, showed slightly different behaviour, with the signal not reaching complete fringe loss before achieving chaos, and also had observable fringes in the external cavity modes. Part of this might be explained by the geometries of the two lasers and how the returning light was coupled back into the laser cavity. In the case of the VCSEL, the light was reinjected into a small lasing cavity via a small circular aperture (∼8 µm diameter). The length of the active region potentially ranged from half a wavelength to several wavelengths, while the DFB had a similarly sized rectangular aperture (∼8 µm by ∼6 µm), and the wave guide (and thus an active region) was in the range of hundreds of µm long (and thus hundreds of wavelengths). We know from similar optical setups that the reinjected spot is on the same order of magnitude as that of laser apertures [69] and has a focus of several wavelengths; thus, the reinjected light in the case of the VCSEL was dominated by the whole lasing cavity; for the DFB with a much larger active region (due to length of the waveguide), it prevented total domination by the optical feedback. This change in coupling also meant that the mapping of the attenuation in the external cavity path and to κ was not exact, and a small offset (in attenuation) between the shared observed phenomena between to the two lasers was present. These observed differences, particularly in the DFB, were dependent on the emitted beam being a perfectly collimated beam (as observed using a shearing plate), and deviations from collimation can produce quite different results.

**Figure 5:**
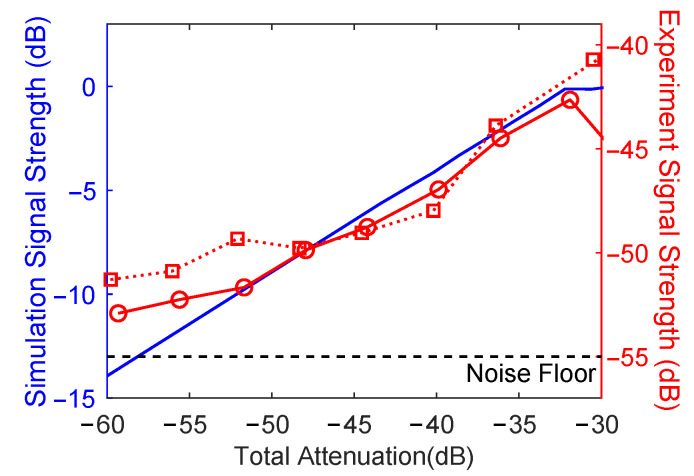
SM signal strength vs. attenuation. Red curves are the experimental result (circles—DFB, squares—VCSEL), and the blue curve is the simulation result.

Similarly, we could generate simulated SM waveforms (in this case, we used parameters to match with the 852 nm DFB laser) with different optical feedback by using Equations (1)–(3) to synthesise the terminal voltage. The AC component of the waveforms was able to generate waveforms sitting in the SM regions where the cavity attenuation was varied from −60 to −30 dB. The laser was observed entering a coherence collapse regime when the attenuation was above ∼−30 dB. The simulated SM signal shown in Figure 4c matched well with the experimental result for each attenuation level at the same regime in Figure 4a,b. Some minor differences appeared in the morphology of the signals in the strong feedback regime; as mentioned earlier, these difference could have been due to spatial coupling effects, which was further reinforced, as these were not present in the 1D model used in the simulations. The normalised signal strength of the SM signal as a function of total attenuation for the same laser was simulated and is shown with a blue curve in Figure 5, which also agreed well with the experimental result, as shown in the red curves in the figure. The shown noise floor was estimated from the average value measured from the two lasers well below the threshold. Both experimental curves seemed to plateau before this point, which may have been due to the peak-to-peak method of estimating signal strength. Figure 4a,b show SM fringes at the highest attenuation level (−60 dB) even though the noise was dominant. This noise was most likely a combination of the effect of feedback, diode junction, laser driver, thermal and amplifier noise, and the effects from light coupling, which was not exactly as expected. This matched well with previously observed effects with THz QCLs, and the SM signal should be able to extracted using techniques that could extract signals from a signal-to-noise ratio of less than 1 (see [24]). Alternatively, using a photodiode to monitor the signal would also help in this regard, as the SM voltage signal tends to have a higher noise floor (typically in the order of ∼10 dB [70]).

**Figure 6:**
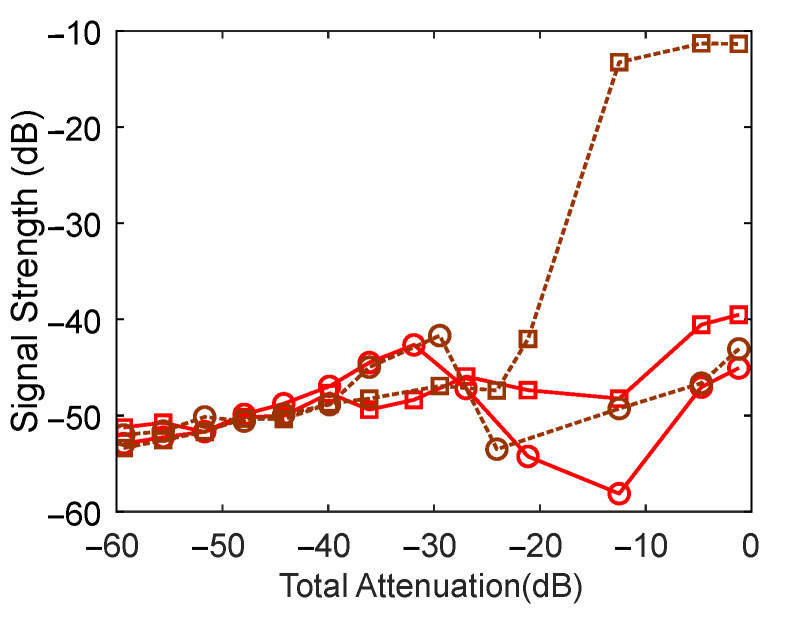
VCSEL (Dashed) and DFB (solid) SM signal strength vs. attenuation. Circles—displacement signal, squares—optically chopped signal.

In terms of the reflection-mode experiments, Figure 6 shows the difference between the displacement amplitude (peak-to-peak) and that of the optically chopped signal (change in reflectivity or β signal). Both signals followed the same trend until strong feedback and markedly deviated once the external cavity modes had been established.

**Figure 7:**
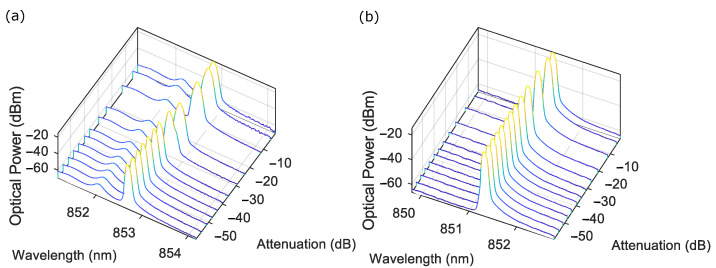
Laser spectra for different feedback levels (attenuation) for a static target. (**a**) VCSEL spectrum at a bias current of 7 mA and laser temperature of 35 ${}^{\circ }$C. (**b**) DFB spectrum at a bias current of 70 mA and laser temperature of 25 ${}^{\circ }$C.

Similarly, the spectra of the two lasers showed a similar behaviour (Figure 7), with slight red shifts with increasing feedback, and the slight broadening and enhancement of side modes as strong feedback was reached (the 850 nm VCSEL actually lases at ∼853 nm, and the 852 nm DFB lases at ∼851.5 nm). Chaos and external cavity modes showed large amounts of broadening, which may have actually been flipping between two or more closely spaced modes that could not be easily captured by the slow sweep rate of the optical spectrum analyser. There was also a large step in the red shift going from strong to chaos and external cavity modes, suggesting (not surprisingly) a significant change in the laser operation at these points.

While the initial spectral line widths were limited by the resolution (0.07 nm) (FWHM ∼0.06 nm for both lasers) of the spectral analyser, as we approached strong feedback, the peaks noticeably broadened for both lasers and were quite significant, with both lasers broadening at the FWHM to around ∼0.09 nm (∼37 GHz). We know from the literature that typical line widths for these diodes lasers are in the tens to hundreds of MHz, meaning that the observed broadening was quite large and fit with the numbers reported in [32]. We could also observe the total shift in the dominant mode vs. feedback (∼−60 dB attenuation to ∼0 dB) for each of the lasers with the VCSEL shifting ∼0.135 nm (∼56 GHz) and the DFB shifting ∼0.045 nm (∼18 GHz). This change was mainly observed in the shift from −30 to 0 dB attenuation for both lasers. The DFB was fairly static, while the VCSEL showed a small shift from the moderate to strong feedback regimes [−45 to −30 dB attenuation, ∼0.03 nm (∼12 GHz)].

## 5. Conclusions

We investigated the mapping of classical optical feedback regimes in terms of the optical feedback level and the bias current of two different diode lasers ( VCSEL and DFB, both lasing at around ∼850 nm). Mapping these parameters showed that, while the behaviour of the two lasers was quite similar and predictable in the simulation, for most situations, there were differences in behaviour that could be attributed to the different coupling conditions arising from the different geometries of the lasing apertures of each laser. In the case of the VCSEL, where the reinjected light very much overlapped with the aperture and active region of the laser, we reported very good agreement with the standard rate equation model that had no spatial dependence (particularity in the strong feedback regime with fringe loss). For the DFB with a larger aperture and active region, the laser behaved quite differently in the strong feedback region. We observed that the behaviour at the onset of SM fringes vs.current was also quite different, with the the DFB occurring at the onset of threshold, while the VCSEL was delayed to slightly after the threshold. The two lasers showed similar levels of broadening (at the FWHM of fundamental mode) in the tens of GHz with strong feedback, but with quite different levels of a fundamental wavelength shift with feedback. However, within Feedback Regimes I–III, the performance of the two lasers in terms of size and viable signal was quite similar, even though the architectures and operating conditions were quite different. This indicated that, while different lasers perform, for the most part, similarly to the ideal case presented in simulations, pushing the operating parameters to extremes causes deviations that may have to be considered.

## Data Availability

The data presented in this study are available on request from the corresponding author.

## Appendix A

**Table A1:** Model parameters and values used in Equations (1)–(3).

Symbol	Description	Value / Units
$V_{\mathrm{Terminal}}\left(t\right)$	Laser Terminal voltage	Varies, V
$N\left(t\right)\;,\left(N_{s}\right)$	Carrier density in the laser cavity, (steady-state)	Varies, $m^{-3}$
$N_{i},N_{\mathrm{tr}}$	Intrinsic carrier density and carrier density at transparency	1.8 $\times 10^{-24}\;m^{-3}$
$\varphi_{\mathrm{FB}}$	Phase under optical feedback, $\varphi_{\mathrm{FB}}=\omega \;\tau_{\mathrm{ext}}$	Varies, rad
$\varphi_{s}$	Phase in the absence of optical feedback, $\varphi_{s}=\omega_{\mathrm{th}}\;\tau_{\mathrm{ext}}$	Varies, rad
$v_{g}$	Laser cavity group velocity, $v_{g}=c/n_{g}$	7.138 $\times 10^{7}\;m\cdot s^{-1}$
$n_{\mathrm{in}}\;,L_{\mathrm{in}}$	Refractive index and length of the laser active region	4.2, 250 µm
$\tau_{\mathrm{in}}$	Laser internal cavity round-trip time, $\tau_{\mathrm{in}}=2L_{\mathrm{in}}n_{\mathrm{in}}/v_{g}$	7.005$\times 10^{-12}\;s$
λ	Laser emission wavelength in vacuum	852 nm
ω	Laser mode angular frequency	Varies, rad
$\omega_{\mathrm{th}}$	Laser mode angular frequency in the absence of optical feedback at threshold	$2.21\times 10^{15}\;\mathrm{rad}\cdot s^{-1}$
*I*	Drive current	60 mA
$\tau_{p}$	Photon lifetime in laser cavity	2.768 $\times 10^{-12}\;s$
α	Henry’s linewidth enhancement factor	3
*a*	Differential gain	$0.17\times 10^{-20}\;m^{-2}$
$W_{\mathrm{in}}\;,D_{\mathrm{in}}$	Width and height of the laser active region	2 µm, 80 Å
*V*	Volume of active region in the laser cavity $V=L_{\mathrm{in}}\times W_{\mathrm{in}}\times D_{\mathrm{in}}$	$4\times 10^{-18}\;m^{3}$
$V_{p}$	Effective cavity volume occupied by photons, $V_{p}=V/\Gamma$	$1.25\times 10^{-16}\;m^{3}$
Γ	Optical confinement factor, $\Gamma =V/V_{p}$	0.032
$R_{2}$	Reflectivity of the right laser facet	0.324
$R_{\mathrm{ext}}$	Reflectivity of external target	0.99
ε	Re-injection coupling factor	Varies
κ	Feedback coupling coefficient, $\kappa =\varepsilon \left(1-R_{2}\right)\sqrt{\frac{R_{\mathrm{ext}}}{R_{2}}}$	Varies
$\tilde{\kappa }$	Feedback coupling rate, $\tilde{\kappa }=\kappa /\tau_{\mathrm{in}}$	Varies, $s^{-1}$
*C*	Feedback level/parameter, $C=\tilde{\kappa }\tau_{\mathrm{ext}}\sqrt{1+\alpha^{2}}$	Varies
$n_{\mathrm{ext}}\;,L_{\mathrm{ext}}$	Refractive index and length of the external cavity	1.00, 0.5 m
$\tau_{\mathrm{ext}}$	Round-trip time of the external laser cavity, $\tau_{\mathrm{ext}}=2n_{\mathrm{ext}}L_{\mathrm{ext}}/c$	$3.336\times 10^{-9}\;s$
A,f	Target harmonic displacement amplitude and frequency	λ, 330 Hz
$k_{B}$	Boltzmann’s constant	$1.381\times 10^{-23}\;J\cdot K^{-1}$
*T*	Temperature	300 K
*q*	Charge on the electron	$1.602\times 10^{-19}$ C
*t*	Time	Varies, s
